# Cardiac and Pulmonary Ultrasound for Diagnosing TRALI

**DOI:** 10.1155/2015/362506

**Published:** 2015-10-28

**Authors:** J. I. Alonso-Fernández, J. R. Prieto-Recio, C. García-Bernardo, I. García-Saiz, J. Rico-Feijoo, C. Aldecoa

**Affiliations:** Anesthesiology and Postoperative Critical Care Department, Río Hortega Universitary Hospital, 47009 Valladolid, Spain

## Abstract

Unexpected acute respiratory failure after anesthesia is a diagnostic challenge: residual neuromuscular blockade, bronchial hyperresponsiveness, laryngospasm, atelectasis, aspiration pneumonitis, and other more uncommon causes should be taken into account at diagnosis. Lung ultrasound and echocardiography are diagnostic tools that would provide the differential diagnosis. We report a suspected case of a transfusion related acute lung injury (TRALI) following administration of platelets. The usefulness of lung and cardiac ultrasound is discussed to facilitate the challenging diagnosis of the acute early postoperative respiratory failure.

## 1. Introduction

Respiratory failure which happens unexpectedly after anesthesia is a diagnostic challenge. Several concurrent causes in emergent surgery could explain respiratory distress and postoperative hypoxemia: residual neuromuscular blockade, bronchial hyperresponsiveness, laryngospasm, atelectasis, chemical pneumonitis, and other rarer causes such as heart failure by volume overload, ARDS due to a septic process, pulmonary embolism, or acute respiratory distress due to transfusion reactions.

## 2. Case Report

We report the case of a 56-year-old woman with personal history of smoking, dyslipidemia, hypertension, overweight (BMI 28), and amaurosis fugax episode whereby continued antiplatelet therapy with clopidogrel. The patient came to the emergency room for severe abdominal pain in the right lower quadrant with nausea and vomiting of 24-hour duration. The examination revealed abdominal distension, painful palpation in the right iliac fossa, and decreased bowel sounds.

The woman underwent abdominal ultrasound, which reported appendage of increased caliber with thickened wall, data compatible with uncomplicated acute appendicitis. She entered the surgery department, beginning antibiotic treatment with amoxicillin/clavulanic and deciding urgent open intervention. The preintervention clinical situation was stable from both respiratory and hemodynamic points of view. The laboratory exams and X-ray exams were also normal before the surgery.

It was decided to administer a pool of platelets, obtained by apheresis with a volume of 250 cc in 30 minutes, half an hour before intervention due to platelet dysfunction by clopidogrel. Pharmacological induction of anesthesia with propofol, fentanyl, and rocuronium was performed. Standard monitorization included SpO_2_, ECG, and noninvasive arterial pressure and neuromuscular block monitoring was applied. Intubation was performed without any problems including vomiting or aspiration. After induction elevated airway pressures were evident, without bronchospasm signs in the auscultation, improving after lung recruitment maneuvers. Subsequently she remained stable from both respiratory and hemodynamic points of view during the whole intervention. The surgery lasted 60 minutes. No general peritonitis was observed. Extubation was performed when the Train of Four ratio was higher than 0.9 and the conscious was recovered. After extubation, while staying at the postanesthesia care unit fifteen minutes after the extubation, she presented with progressive dyspnea, cyanosis, and severe agitation which may be related to the hypoxia and necessitated the administration of high-flow oxygen. A lung auscultation showed no rales, rhonchi, or wheezing. She was moved to the Resuscitation Unit where she persisted in need of high concentrations of oxygen. The patient remained hemodynamically stable without tachycardia and in sinus rhythm. No hypothermia and shivering were observed.

**Figure 1:**
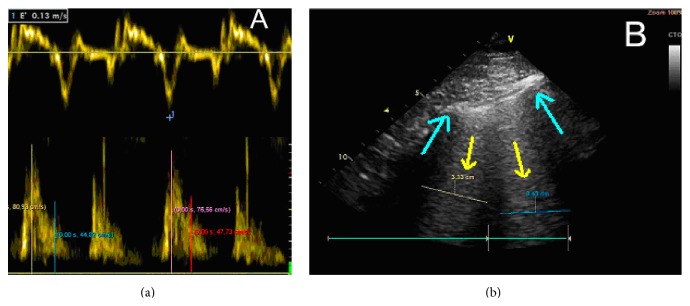
(a) Top: Doppler tissue of the lateral mitral annulus showing the value of *e*′. Bottom: Doppler mitral filling. Together both images show a pattern of normal mitral filling estimate normal filling pressure (value *E*/*e*′ < 8). (b) The picture shows lung ultrasound realized with a sectorial probe in the patient's right chest, between fourth and fifth ribs in midclavicular line. Figure shows presence of vertical B lines > 3 mm (yellow arrows), indicative of pulmonary edema. Blue arrows show rib shadows.

She underwent an urgent echocardiogram by an expert operator showing cardiac chambers of normal size, preserved systolic function, and normal pattern of relaxation filling mitral *E*/*e*′ = 6.2 (lateral measures) and the tissue Doppler shows *a*′ minor compared to *e*′ (see Figure 1). Also the presence of significant valvular and pericardial effusion was discarded. In the thoracic ultrasound the presence of B lines and areas of subpleural condensation in both hemithoraces were shown (see Figure 1). An urgent chest radiograph was requested showing the presence of bilateral pulmonary infiltrates, not present previously on admission. Noninvasive mechanical ventilation was established after the TEE and the results were observed. Arterial blood and gas analysis were repeated, confirming the existence of moderate respiratory failure (PaO_2_/FiO_2_ = 84/0.6 = 140).

Faced with the possibility of having platelet transfusion reaction, it was reported to the Hematology Department that dismissed the remaining blood components from the same donor. Procalcitonin and lactate were also requested, showing values within normal ranges. Diuretic therapy was initiated without improvement of pulmonary infiltrates in the following hours. In serial radiographs in 48 hours, the pulmonary infiltrates persisted. She remained hemodynamically stable during the next three days. The antibiotic treatment was discontinued on the third day of admission due to lack of fever and leukocytosis. From a respiratory point of view she needed noninvasive ventilation with pressure support of 15 cmH_2_O. The patient was discharged with supplemental oxygen by nasal cannula on the fifth day of admission in the Resuscitation Unit. X-ray and lung ultrasound at discharging were normal.

## 3. Discussion

TRALI is a clinical syndrome presented as acute hypoxemia and noncardiogenic pulmonary edema during or after a transfusion of blood products. There is no single definition of this process, but in 2004 the Canadian Blood Service and Hema-Quebec proposed in its consensus conference some criteria [1] that have been widely accepted. Its incidence is not well established. Thus, Silliman et al. [2] establish the existence of TRALI in 1 over 1323 blood product transfusions. Predisposition also seems to be different depending on the type of blood product infused, from highest to lowest risk: platelet transfusion of whole blood, apheresis platelets, packed red cells, and fresh frozen plasma. Its pathophysiology is not completely enlightened [3] and could be explained by a dual mechanism, with the presence of leukocyte immune antibodies or a cytokine liberation due to a preexisting lung damage and subsequent migration of neutrophils, causing that pulmonary capillary injury.

The clinical presentation includes dyspnea, tachypnea, and hypoxemia as cardinal symptoms. Fever, tachycardia, hypotension, and even hypertension may also be present. There is no laboratory test to confirm the diagnosis of TRALI.

The differential diagnosis of a patient who suddenly develops respiratory failure after a transfusion of blood products should include hemodynamic overload, anaphylactic reaction, bacterial contamination of blood products transfused, and hemolytic transfusion reaction.

In the reported case, the differential diagnosis of hemodynamic overload was performed using echocardiographic assessment, including both systolic function as noninvasive estimation of filling pressure by *E*/*e*′.

Transthoracic two-dimensional echocardiography has become an essential tool for the confirmation of ARDS, after following the redefinition of Berlin. The morphological study will allow us to analyze the size, morphology, and global and segmental contraction. It allows discarding dilated cardiomyopathy, hypertrophic cardiomyopathy, ventricular dysfunction, or acute stress cardiomyopathy. Application of Doppler will allow us to assess valvular dysfunction and a more detailed assessment of the hemodynamic status and diastolic function [4]. The “early mitral flow peak velocity to early diastolic mitral annulus displacement velocity” (*E*/*e*′) ratio correlates closely with left ventricular end-diastolic pressures (LVEDP). The mitral filling pattern with *E*/*e*′ is a method to estimate the right ventricular filling. Values above 15 have been correlated with high pulmonary wedge pressure and values below 9 have been correlated with normal one [5]. Adding pulmonary venous flow to *E*/*e*′ ratio may facilitate LVEDP assessment. To improve the accuracy of *E*/*e*′ to assess the left ventricular pressure and discard a pseudonormal pattern, the use of the values of tissue Doppler has been proposed. When the relationship in *e*′/*a*′ is higher than 1, a normal pattern is present. However, the assessment of pulmonary venous flow using a transthoracic approach is difficult.

The morphology and function of the right ventricle can also rule out the presence of acute or chronic cor pulmonale. Overall, the information provided by echocardiography allowed excluding cardiogenic pulmonary edema as the primary cause of respiratory failure.

Lung ultrasound is a supplement to the information provided by echocardiography. The pulmonary ultrasound is able to define numerous causes that alter pulmonary function [6]. In the ultrasound technique, lung, ribs, spine, and lung air act as barriers to ultrasounds, causing artifacts that we recognize and interpret for a correct diagnosis. The altered lung presents a change in the air/intrathoracic liquid ratio, with this liquid being either alveolar or interstitial edema or also blood, mucus, pus, or increased cellularity. The exploration is done in the supine position, allowing easy anterolateral approach, and according to the BLUE protocol [7] 3 points in each hemithorax are enough to draw conclusions. Firstly, the presence of pleural sliding sign rules out pneumothorax as a cause of respiratory failure. The presence of B lines, vertical lines starting from the pleural line and extending in depth, with variations in size synchronized with the respiratory cycle is suggestive of an alveolar interstitial syndrome, which means cardiogenic or noncardiogenic pulmonary edema. Multiple B lines 7 mm apart are caused by thickened interlobular septa characterizing interstitial edema [8]. In contrast, B lines 3 mm or less apart are caused by “ground-glass” areas characterizing alveolar edema [9]. The presence of subpleural parenchyma with patchy consolidations and lines B is more suggestive of ARDS heart failure, as what happened in our case [10]. The learning curve for B line assessment is short [11] and this method is reproducible without any variability [12]. Thus, the information that has been given by pulmonary ultrasound outdoes chest X-ray [13]. The data supplied by echocardiography on systolic and diastolic function help to define better the origin of pulmonary disorders.

However, although this approach is useful in most cases, an area of uncertainty exists when *E*/*e*′ values are between 9 and 15 and lung ultrasound does not show any data of parenchymal damage. In such cases, advanced echocardiographic methods [14] for estimating pulmonary wedge pressure, further evaluations, or invasive measurements may be needed.

The absence of rash, urticaria, or angioedema during administration of platelets and the absence of clinical and laboratory data (procalcitonin) in severe sepsis and pre-post intervention also strengthens the diagnosis of acute lung injury associated with transfusion (TRALI).

In conclusion, while managing a patient who has received some type of blood product, the presence of respiratory failure requires a differential diagnosis with TRALI. The reversal of antiplatelet effect through platelet transfusion should be individualized on urgent interventions. Echocardiography together with thoracic ultrasound facilitates the differential diagnosis of respiratory failure in the surgical critical patient.
